# Fabrication of Novel Omeprazole-Based Chitosan Coated Nanoemulgel Formulation for Potential Anti-Microbia; In Vitro and Ex Vivo Characterizations

**DOI:** 10.3390/polym15051298

**Published:** 2023-03-04

**Authors:** Irshad Ullah, Aiyeshah Alhodaib, Iffat Naz, Waqar Ahmad, Hidayat Ullah, Adnan Amin, Asif Nawaz

**Affiliations:** 1 Department of Pharmacy, University of Swabi, Swabi 23430, Pakistan; 2 Department of Physics, College of Science, Qassim University, Buraydah 51452, Saudi Arabia; 3 Department of Biology, Science Unit, Deanship of Educational Services, Qassim University, Buraydah 51452, Saudi Arabia; 4 Institute of Chemical Sciences, Gomal University, Dera Ismail Khan 29220, Pakistan; 5 Gomal Centre of Pharmaceutical Sciences, Faculty of Pharmacy, Gomal University, Dera Ismail Khan 29220, Pakistan

**Keywords:** Omeprazole, nanoemulgel, chitosan, ex-vivo permeation, minimum inhibitory concentration, drug design, antimicrobial activity

## Abstract

Infectious diseases remain inevitable factors for high mortality and morbidity rate in the modern world to date. Repurposing is a novel approach to drug development has become an intriguing research topic in the literature. Omeprazole is one of the top ten proton pump inhibitors prescribed in the USA. The literature suggests that no reports based on omeprazole anti-microbial actions have been discovered to date. This study entails the potential of omeprazole to treat skin and soft tissue infections based on the literature’s evident anti-microbial effects. To get a skin-friendly formulation, a chitosan-coated omeprazole-loaded nanoemulgel formulation was fabricated using olive oil, carbopol 940, Tween 80, Span 80, and triethanolamine by high-speed homogenization technique. The optimized formulation was physicochemically characterized for zeta potential, size distribution, pH, drug content, entrapment efficiency, viscosity, spreadability, extrudability, in-vitro drug release, ex-vivo permeation analysis, and minimum inhibitory concentration determination. The FTIR analysis indicated that there was no incompatibility between the drug and formulation excipients. The optimized formulation exhibited particle size, PDI, zeta potential, drug content, and entrapment efficiency of 369.7 ± 8.77 nm, 0.316, −15.3 ± 6.7 mV, 90.92 ± 1.37% and 78.23 ± 3.76%, respectively. In-vitro release and ex-vivo permeation data of optimized formulation showed 82.16% and 72.21 ± 1.71 μg/cm^2^, respectively. The results of minimum inhibitory concentration (1.25 mg/mL) against selected bacterial strains were satisfactory, suggesting a successful treatment approach for the topical application of omeprazole to treat microbial infections. Furthermore, chitosan coating synergistically increases the antibacterial activity of the drug.

## 1. Introduction

One of the top ten PPIs prescribed in the USA is omeprazole (OMP) [1]. It is the first clinically useful substituted derivative of benzimidazole and prevents gastric acid secretion by inhibiting parietal cells’ H+/K+-ATPase in a non-competitive manner. OMP is considerably broken down in the stomach and is unstable when exposed to an acidic medium. It is also susceptible to heat, light, and humidity [2]. Additionally, the drug’s bioavailability is just 30–40% and has a short half-life of 0.5–1 h [3]. OMP possesses a shorter half-life, decomposition in an acidic environment, and additionally the dearth of an appropriate transdermal drug delivery system to highlight its anti-microbial effects [4]. Repositioning and repurposing an already-available active pharmacological ingredient for a different use is the basis of drug repositioning. This approach has a number of disadvantages and presents certain difficulties, but it also has numerous benefits, such as the current attrition in novel drug discovery can effectively be surmounted by employing this strategy [5]. This approach entails identifying novel applications for drugs that have already received clinical approval and have a well-established structure, safety, and toxicity profile. Since they can be implemented quickly, efficiently, and at a lesser cost than developing a new drug moiety, therefore they can be repurposed in the effective management of various pandemics and emerging diseases. Drug repurposing has played a pivotal role in the effective treatment of infectious disorders, as evidenced by recent research on preeminent bacterial infections and successful precedents, including anti-hypertensives [6], anti-inflammatory agents [7], statins [8], anti-depressants [9], anti-histamines [10], benzodiazepines [11], and alcohol aversive agents [12]. Thus, the literature is replete with studies designed to highlight an existing drug’s benefit in additional indications.

Nanoemulsions are kinetically stable dispersions that have oil and water domain interfaces made of surfactants. These formulations allow the localization of hydrophobic drugs in various dermal appendages as well as reservoirs. This strategy also emulsifies both lipophilic as well as hydrophilic drugs while using less surfactant concentration [13]. The most crucial aspect is that this drug delivery system can be formulated readily into nanoemulgel by the addition of gelling agents, to guarantee optimal therapeutic dosage administration through the skin [14]. In nanoemulgels, nanoemulsion globules are entrapped by a three-dimensional network formed by polymers or gelling agents. Additionally, it features nano-sized particles that enable quick delivery and permeation of the active drug and a dual-release control mechanism (hydrogel and nanoemulsion) [15]. 

The literature also suggested that OMP is widely used in the treatment of *H. pylori,* but the activity was not specific to Helicobacter spp., since other bacteria bound omeprazole and were susceptible to different degrees. The further suggested that omeprazole was found to bind to *H. pylori* as well as to other gram-negative and gram-positive bacteria [16]. 

Chitosan was first discovered in 1811, in a mushroom by French researcher Henri Braconnot [17]. Afterward, researchers found it in alternative sources, i.e., from fungi, aquatic species, and other terrestrial living organisms [18]. There are two extraction methods for chitosan, including, chemical treatment and enzymatic treatment. The process of chemical deacetylation is used to obtain chitosan from crustaceans and the method involves demineralization, deproteinization, decolorization, and deacetylation [19]. In the second method, chitosan is enzymatically produced by using chitin deacetylase. Chitin deacetylase can be obtained from various fungal, bacterial, and some species of insects [20]. Chitosan is a semi-crystalline polysaccharide, containing N-acetyl-d-glucosamine and d-glucosamine residues arranged in linear dimension. It is cationic in nature, due to the presence of an amino group (–NH_2_) in its structure [21]. This positive charge helps the formation of the extracellular matrix by attracting negatively charged molecules, such as proteoglycans [22]. In addition, the hydroxyl group (–OH) is also present in the structure and attracts positively charged molecules to enhance the bonding [23]. In addition to electrostatic attractions, these functional groups help in the modification of chitosan, thus improving its mechanical and biological properties to bring novel functional properties and promising practical applications [24]. Chitosan is well known for its action against a wide-range of microbes due to its poly-cationic structure which makes it a promising antimicrobial candidate [25]. In its structure, the positively charged amino group interacts electrostatically with the negatively charged microbial membranes, resulting in the leakage of cellular contents and finally cell death [26]. Chitosan also acts as an antioxidant agent by forming stable radicals with its functional groups [27]. Chitosan also acts as an anti-inflammatory agent [28]. It has been suggested that the anti-inflammatory activity of chitosan is due to the presence of charged moieties in its structure, which negatively regulates the pro-inflammatory reactions [29]. Fungal-sourced chitosan can be used in food, pharmaceutical, or biomedical applications for different applications, for example, as an antimicrobial agent, coating material, water purification, or bio-pesticide [30].

In order to develop an omeprazole (OMP) loaded topical dosage form to treat skin and soft tissue infections, with proven evidence of exhibiting anti-microbial effects, the current study demonstrates the fabrication, optimization, and characterization of nanoemulgel formulation loaded with OMP. By employing a high-speed homogenization process and chitosan as the backbone, a nanoemulgel formulation of OMP was prepared. Omeprazole is mostly used in the treatment of *H. pylori* and no further studies were carried out for its use as a topical agent for treating skin microbial infections. The current study depicted that omeprazole showed significant results in treating microbial infections. Further studies should also be recommended to investigate the antimicrobial effect of omeprazole. 

## 2. Materials

Omeprazole in pure form was obtained from Ferozsons Laboratories, Nowshehra, Pakistan. Olive oil (Marhaba Laboratories, Punjab, Pakistan), Carbopol 940 (Dow Chemical Company, Midland, MI, USA), Tween 80, Span 80, DMSO (Sigma Aldrich, St. Louis, MO, USA), and triethanolamine (BDH, Poole, UK) were used in the preparation of nanoemulsion and nanoemulgel. All the excipients and oils used in the formulation of omeprazole-loaded nanoemulgel formulations were of analytical grade and in pure form.

### 2.1. Formulation and Optimization of OMP Loaded Nanoemulgel

#### 2.1.1. Nanoemulsion Preparation

**Figure 1:**
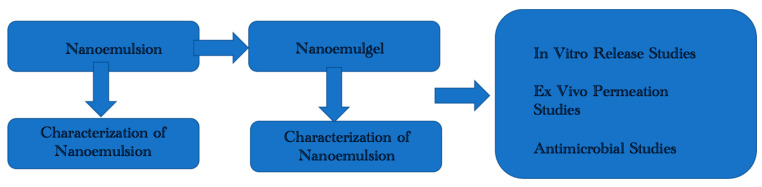
Scheme of the experimental procedure.

The scheme of the experimental procedure was shown in Figure 1. OMP-loaded nanoemulsion was fabricated using a high-pressure homogenization approach in accordance with a previously described, but slightly modified, procedure. The oily phase (A) was constituted by combining olive oil, Span 80, and drug. Likewise, the aqueous phase (B) was obtained by dissolving Tween 80 in distilled water. Both phases were maintained at a temperature of 60 ± 5 °C for 30 min by employing a water bath (Precision Scientific, Model 181, Japan). Chitosan solution (0.1% *w*/*w*) was prepared in 1% *v*/*v* acetic acid solution and then transferred to the aqueous phase. The oil phase was introduced dropwise to the aqueous phase after both phases had been heated, and the mixture was stirred for 10 min at 1500 rpm. The mixture was cooled gradually, and for 10 min, the stirrer’s speed was dropped to 1000 rpm. The stirrer’s speed was once more increased to 1500 rpm for 10 min to ensure appropriate mixing and the formation of a homogenous emulsion. After allowing the emulsion to cool, a high-speed homogenizer (Daihan, Korea) was used for 10 min at 15,000 rpm. Different formulations with varied constituent concentrations were developed for optimization purposes, as evident in Table 1.

#### 2.1.2. Nanoemulsion Stability

Prior to combining nanoemulsion formulations with carbopol gelling solution, they were subjected to organoleptic evaluation. To achieve this objective, their color shift, uniformity, and phase separation were all noted. Samples of each formulation were held at various temperatures i.e., 8 °C, 25 °C, 40 °C, and 40 °C ± 75%, relative humidity, for 28 days in order to evaluate their thermostability and storage potential [31].

#### 2.1.3. Carbopol Gelling Solution Preparation

In this process, 1 g of carbopol was dispersed in 100 mL of distilled water to obtain carbopol gel, which was then incorporated into nanoemulsion formulations after keeping the gel solution overnight.

#### 2.1.4. Nanoemulgel Preparation

Nanoemulsion was thoroughly mixed with carbopol gelling solution in a ratio of 1:1 and continuously stirred for 15 min. As a result, a homogenous semisolid nanoemulgel formulation was prepared. Finally, triethanolamine was added to the nanoemulgel formulation for adjusting its pH in the range of human skin pH (4.5–5.5), to avoid any toxicity [24].

### 2.2. Characterization of Optimized Nanoemulsion & Nanoemulgel Formulations

#### 2.2.1. Thermodynamic Stability Studies

Different thermodynamic stability tests such as centrifugation, heating cooling cycles, and freeze-thaw cycles, were carried out for optimized omeprazole-loaded nanoemulsion formulation (OMP3NE) and omeprazole-loaded nanoemulsion gel formulation (OMP3NEG) to investigate their behavior under stressed environments. These tests were conducted in accordance with ICH recommendations and previous studies [25].

#### 2.2.2. ATR-FTIR Analysis

To ascertain the compatibility profile between the formulation components and polymer, the drug, carbopol powder, optimized nanoemulsion, and nanoemulgel formulations were evaluated via ATR-FTIR spectroscopy (Perkin-Elmer, Buckinghamshire, UK). Samples of formulation components and polymer were placed over a diamond crystal and pressure was applied by means of a knob. Each sample spectrum was taken in a range of 400–4000 cm^−1^ wavenumber, and triplicate readings were recorded [26].

#### 2.2.3. Size, Zeta Potential, and Polydispersity Index (PDI)

This experiment was run to determine droplet size, zeta potential, and PDI of optimized nanoemulsion and nanoemulgel formulations. Zeta sizer and helium-neon laser (Malvern Instruments, Worcestershire, UK) were employed for this purpose. The optimized formulation was diluted with the help of deionized water in the ratio of 1:9 and the mixture was thoroughly stirred for 3 min. The experiment was run in triplicates, and the results were shown as mean ± SD [27].

#### 2.2.4. pH Determination

The pH of freshly prepared optimized OMP-loaded nanoemulsion and nanoemulgel formulations was determined with the help of a digital pH meter, by taking the samples after 12 h, 24 h, and 7, 14, and 28 days [28].

#### 2.2.5. Surface Morphology Analysis

The morphological attributes of optimized nanoemulgel formulation (OMP3NEG) were demonstrated with the help of TEM (Oregon, USA). The nanoemulgel formulation was diluted 10 times and fixed on a copper grid, dried, and imaged [29].

#### 2.2.6. Drug Content

The drug content of optimized nano formulations was evaluated by combining 2 g of each formulation with 10 mL of phosphate buffer solution (pH 5.5). After that, the sample was centrifuged for 30 min at 14,000 rpm. Then, a sample of the clear liquid was obtained for spectrophotometric analysis at a maximum wavelength of 324 nm by utilizing a UV-spectrophotometer (UV-2600i, Shimadzu, Kyoto, Japan). Following the conversion of the observed absorbance to its corresponding concentration by employing a calibration curve, the precise concentration of the drug in the formulation was estimated [30].

#### 2.2.7. Entrapment Efficiency

For the determination of entrapment efficiency, the optimized nanoemulsion and nanoemulgel formulations were dissolved in methanol and then subjected to centrifugation for 15 min at a temperature of 20 °C in a cooling microfuge. The collection and filtration of supernatant were carried out after centrifugation. Then, dilution of the stock solution was performed by taking its 0.2 mL volume in 1 mL of water. The amount of drug present in un-entrapped form was evaluated by UV-spectrophotometric analysis (UV-2600i, Shimadzu, Kyoto, Japan) at λmax of 324 nm. By deducting un-entrapped drugs from the total drug, drug entrapment was calculated which was then used for the evaluation of entrapment efficiency.

#### 2.2.8. Viscosity Determination

The viscosities of optimized drug-loaded nano formulations were estimated by means of a viscometer (NDJ, 8S, Republic of Korea) at various temperatures (8, 25, and 40 °C) after taking the samples at an interval of 0, 1, 2, 7, 14, and 28 days [32].

#### 2.2.9. Spreadability and Extrudability Estimation

Based on a previously published method [33], the spreadability and extrudability of the optimized nanoemulgel formulation were examined. In a nutshell, 1 g of nanoemulgel was used to test spreadability, and 10 g to test extrudability. The experiment was repeated three times and then averaged. For spreadability estimation, the Drag and Slip apparatus was employed. In this technique, an accurate amount of sample was placed on a glass slide, and another glass slide was placed over it so that the sample between the two glass slides is sandwiched. It was followed by the placement of 100 g weight over the glass slides. The sample was compressed and formed a homogenous thin coat and in addition, excessive removal of the sample was also achieved. The bottom glass slide was securely tightened to the platform with minimal damage, but only the top glass slide was able to be easily withdrawn after being tied with a 20 g weight. The time it took to slide the upper glass over a distance of 7.50 cm across the thin coating on the bottom slide was used to calculate spreadability by the following equation:

S = M × L/T(1)

where S represents spreadability, M represents weight placed on the upper glass slide, L represents the length of the slide, and T represents the time taken for the separation of slides.

For the determination of the extrudability of optimized nanoemulgel formulation, the amount of formulation extruding from collapsible tubes was assessed. Collapsible tubes were filled with a weighed amount of the formulation. After that, the weight required to extrude a 1 cm formulation ribbon from the collapsible tubes was used to calculate the extrudability. In general, the greater the amount of nanoemulgel extruded, the greater the extrudability. The experiment was performed in triplicates, and the following equation was utilized to calculate extrudability [34]:

E = M/A(2)

where E represents extrudability, M represents the weight needed to extrude gel from a collapsible tube, and A represents the area.

### 2.3. In-Vitro Drug Release Analysis

An in-vitro drug release experiment was carried out in accordance with the earlier investigation. Franz diffusion cell (IPS Technologies, Maharashtra, India), having donor and receptor compartments with capacities of 6 and 3 mL, respectively, was employed for this purpose. The stirring speed was kept at 300 rpm and the temperature at 37 ± 1 °C, before adding the optimized nanoemulsion and nanoemulgel samples. In order to separate the donor and receptor compartments, a Tuffryn membrane (Sartorius, Göttingen, Germany) was clamped. Sodium acetate buffer (pH 5.5) was used to fill the receptor compartments, and formulation (2 g) was put into the donor compartment. After specified time intervals of 0, 1, 2, 4, 8, and 12 h, approximately 2 mL of samples were withdrawn from the receptor compartment by syringe, which was then replenished with fresh buffer solution in order to maintain the sink environment. After the collection of samples, the amount of drug present in the samples was evaluated by subjecting them to UV-spectrophotometric analysis (UV-2600i, Shimadzu, Kyoto, Japan) by taking absorbance at λmax of 324 nm [35].

### 2.4. Ex-Vivo Analysis

#### 2.4.1. Ethics

The Gomal University Ethical Review Board, Dera Ismail Khan, Pakistan, authorized this research under reference number 116/ERB/GU dated 26 February 2022. All the tests were carried out in accordance with appropriate standards.

#### 2.4.2. Rabbit Skin Preparation

According to a previous study [36], the preparation of rabbit skin was conducted for ex-vivo permeation. A male rabbit weighing about 1.5 kg was employed in this experiment which was obtained from in vivo research facility of Gomal University D.I. Khan, Pakistan. The removal of hairs from the dorsal surface of rabbit skin was accomplished by means of hair removal cream. The method of cervical dislocation was employed for the sacrifice of the rabbit. A surgical blade was utilized for the excision of skin, which was then washed and defatted. The skin was then packed in aluminum foil and kept in the refrigerator for storage. It was placed in warm water for an hour on the day of the experiment before being clamped in the Franz diffusion cell for the analysis of ex-vivo permeation.

#### 2.4.3. Ex-Vivo Permeation

Ex-vivo drug permeation testing was conducted in accordance with earlier research. The previously prepared skin was fixed in place between the donor and receptor compartments. Phosphate buffer solution (pH 7.4) was added to the receptor compartment, and 2 g of the prepared sample was added to the donor compartment. After specified time intervals of 1, 2, 4, 8, 16, and 24 h, approximately 2 mL of samples were withdrawn from the receptor compartment by syringe, which was then replenished with fresh buffer solution in order to maintain the sink environment. After the collection of samples, the amount of drug present in the samples was evaluated by subjecting them to UV-spectrophotometric analysis (UV-2600i, Shimadzu, Japan) by taking absorbance at λmax of 324 nm [37].

### 2.5. Anti-Microbial Assay

In this study, many bacterial strains were used. *Escherichia coli*, *Klebsiella pneumoniae*, *Pseudomonas aeruginosa*, and *Staphylococcus aureus* were the strains. These strains were chosen because of their recurrence in damaged skin. Since Mueller-Hinton broth supports the growth of the majority of bacteria, it is the most popular medium for the determination of minimum inhibitory concentrations [33] for antibiotics. Individual bacterial strains were grown on agar and then incubated at 35 ± 2 °C for 16–20 h. The inoculum was made using four or five colonies of pure cultures grown on agar, or roughly 5 × 10^5^ cfu/mL. In order to remove any residues of agar and to ensure the complete separation of colonized bacteria, the colonies were briefly brushed with a loop followed by their transfer to test tubes for dilution with 2 mL of saline. Then, 250 μL of the bacterial suspensions were transferred to additional test tubes of the same size and shape that had been pre-filled with 750 μL of Mueller-Hinton broth. Once the bacteria attained turbidity comparable to a 0.5 McFarland standard, the broth was incubated at 35 ± 2 °C. Utilizing a UV spectrophotometer (UV-2600i, Shimadzu, Japan) set to a maximum 324 nm wavelength, the degree of turbidity was determined. Increasing turbidity or dilution with more broth can be utilized to adjust the optimum turbidity level by the transfer of saline infected with bacteria.

By using the broth dilution procedure, MIC was established. Before placing the test samples to be examined for MIC in the very top well of each column of the 96-well microplate, 50 μL of nutrient broth was first added to each well. After that, it was successively diluted downward. Finally, selected bacterial strains were introduced to each well, followed by a 24-h incubation period at 37 °C. In 96 microplates, 0.015% resazurin solution was injected to track the color shift [38]. Afterward, the turbidity of 96-well microplates was measured on a Microwell plate reader (Hippo HPP 96, Biosan, Lehi, UT, USA). The lowest concentration showing the least turbidity was considered as minimum inhibitory concentration [33].

### 2.6. Stability Studies

Stability analysis was carried out according to ICH guidelines. The prepared nanoemulgel formulation (OMP3NEG) was evaluated for physical and chemical stability studies for a time period of 3 months. The optimized nanoemulgel formulation (OMP3NEG) was put into a glass vial and held for 3 months at 4 ± 2 °C and 40 ± 2 °C. Following that, the appearance, as well as clarity of optimized formulation, was examined by critical visual inspection of the vial. Moreover, particle size, PDI, zeta potential, pH, phase separation, and drug content was also evaluated for OMP3NEG formulation [39].

### 2.7. Statistical Analysis

Student’s *t*-test and one-way ANOVA were utilized as statistical tools, by employing IBM SPSS version 20. All the experiments were carried out in triplicates and results were expressed as mean ± SD.

## 3. Results & Discussion

### 3.1. Thermodynamic Stability Studies

Blank NE, optimized OMP-loaded nanoemulsion (OMP3NE), and nanoemulgel formulations were maintained at various storage environments i.e., 8, 25, and 40 °C, for a time period of 60 days. They were critically investigated for color shift, homogeneity, phase separation, cracking, and liquefaction after regular time intervals. All the freshly prepared formulations passed the required thermodynamic stability tests and were found to be light brown in appearance, and homogenous, and no evidence of cracking or phase separation was observed after being centrifuged at 5000 and 10,000 rpm, as evident in Table 2.

### 3.2. ATR-FTIR Analysis (Drug Excipient Compatibility)

**Figure 2:**
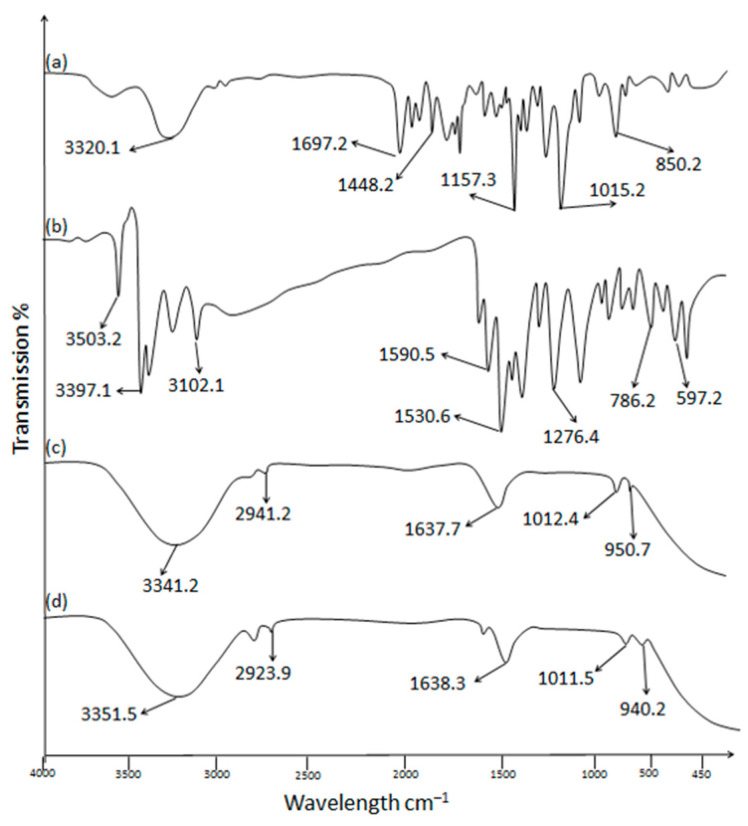
ATR-FTIR spectra of (**a**) OMP (**b**) Chitosan (**c**) OMP3NE (**d**) OMP3NEG.

ATR-FTIR spectra with characteristic peaks of OMP, carbopol 940, OMP3NE, and OMP3NEG are illustrated in Figure 2. The FTIR spectra of pure OMP showed several characteristic bands, 3320.1 cm^−1^ O-H stretching alcohol, 1697.2 cm^−1^ C=O stretching, 1448.2 cm^−1^ C-H bending, 1157.3 cm^−1^ C-O stretching, 1015.2 cm^−1^ and 802.2 cm^−1^ C=C bending respectively. The FTIR spectra of pure chitosan showed several characteristics peaks at 3503.2 cm^−1^, 3397.1 cm^−1^, 3102.1 cm^−1^, 1590.5 cm^−1^, 1530.6 cm^−1^, 1276.4 cm^−1^, 786.2 cm^−1^ and 597.2 cm^−1^ [40]. The formulation OMP3NE showed the characteristics bands at 3341.2 cm^−1^, 2941.2 cm^−1^, 1637.7 cm^−1^, 1012.4 cm^−1^ and 950.7 cm^−1^, while the formulation OMP3NEG showed its characteristics peaks at 3351.5 cm^−1^, 2923.9 cm^−1^, 1638.3 cm^−1^, 1011.5 cm^−1^ and 940.2 cm^−1^ respectively. By looking at the FTIR spectra of the optimized nanoemulsion and nanoemulgel formulations, it can be inferred that, in contrast to the polymer and drug peaks, no new peaks disappeared or appeared in the formulations, indicating that there had been no chemical interactions between the drug and polymer and that OME had been successfully encapsulated in the nanoemulgel formulation.

### 3.3. Size, PDI, and Zeta Potential

**Figure 3:**
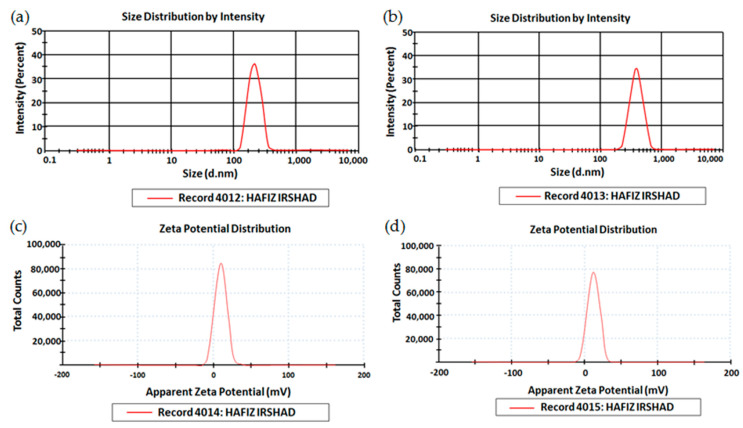
Zeta size of (**a**), OMP3NE and (**b**), OMP3NEG, Zeta potential of (**c**), OMP3NE (**d**), OMP3NEG.

The results of particle size, size distribution, and zeta potential of optimized nano formulations (OMP3NE and OMP3NEG) are shown in Figure 3. The formulations employed for transdermal drug administration are significantly influenced by their droplet size. The droplet size and particle size distribution have an impact on a number of promising factors, including drug release, drug penetration, and bio-distribution. Small droplet size is responsible for greater surface area, resulting in enhanced drug release in an aqueous environment and improved drug absorption [34]. It has been hypothesized that the inclusion of drug particles causes them to interact with the microstructure of the system, reducing globule size, particularly if the drug exhibits an amphiphilic behavior [35]. PDI ensures the homogeneity of globule size within the formulation. The formulation contains globules of a homogenous and uniform size if the PDI value is less than 0.5 [36]. Zeta potential values of optimized drug-loaded nanoemulsion and nanoemulgel formulations were optimum (−11.2 ± 5.4 mV and −15.3 ± 6.7 mV), demonstrating the system’s physical stability. The zeta value describes electrostatic repulsion between particles. The stability of preparations with no evidence of aggregation is guaranteed by zeta potential values greater than ± 30 mV [37]. Higher zeta potential values confirmed the electrostatic repelling associations between oil droplets, which prevented coalescence phenomena and produced an appropriately stable and homogenous dispersion. Overall, the system is negatively charged due to the presence of free fatty acids which impart a negative charge to oil globules [38].

### 3.4. pH Determination

The pH values of optimized nanoemulsion and nanoemulgel formulations were found to be within acceptable human skin pH range (5–6), at various time intervals i.e., 12, 24 h, 7, 14, and 28 days. This is appropriate for use with transdermal drug delivery. The pH is a crucial aspect, and its value must be in the range of 5–6 to avoid skin toxicity [39]. A statistically insignificant difference (Student’s *t*-test, *p* > 0.05) was observed in pH values for all formulations.

### 3.5. Drug Content Determination

The percent of drug content can be used to validate the homogenous drug distribution within formulation [40]. The percent drug content in optimized nanoemulsion, as well as nanoemulgel formulations, are shown in Table 3. The drug concentration in OMP3NE and OMP3NEG according to spectrophotometric analysis was insignificantly different (*p* > 0.05) and was shown to be 93.43 ± 1.66% and 90.92 ± 1.37%, respectively. These values indicate optimum drug loading capability in nanoemulsion as well as nanoemulgel formulations, which is a prerequisite in case of their development. The drug content results showed that it was within the official range of 100 ± 10%, as recommended by the United States Pharmacopeia (USP).

### 3.6. Entrapment Efficiency

The effectiveness of a nanocarrier in holding the active moiety and ensuring its adequate targeted delivery is determined by its entrapment efficiency. Various aspects of the formulation such as preparation technique, excipients employed in the formulation, and the physicochemical characteristics of active moieties entrapped in vesicles are all critical variables that can significantly affect entrapment efficiency. Additionally, as the concentration of active components rises, particle size increases, decreasing the entrapment efficiency of nano formulations [41]. The centrifugation technique was used to determine the entrapment efficiency of developed optimized nanoemulsion and nanoemulgel formulations. The entrapment efficiency of OMP3NE as well as OMP3NEG are displayed in Table 3 and are found to be 81.36 ± 1.98 and 78.23 ± 3.76%, respectively. The homogeneity, as well as the high entrapment efficiency of the system, is impacted by the greater solubility of the drug in the oily phase and the drug’s excipients’ compatibility profile.

### 3.7. Surface Morphology Analysis

**Figure 4:**
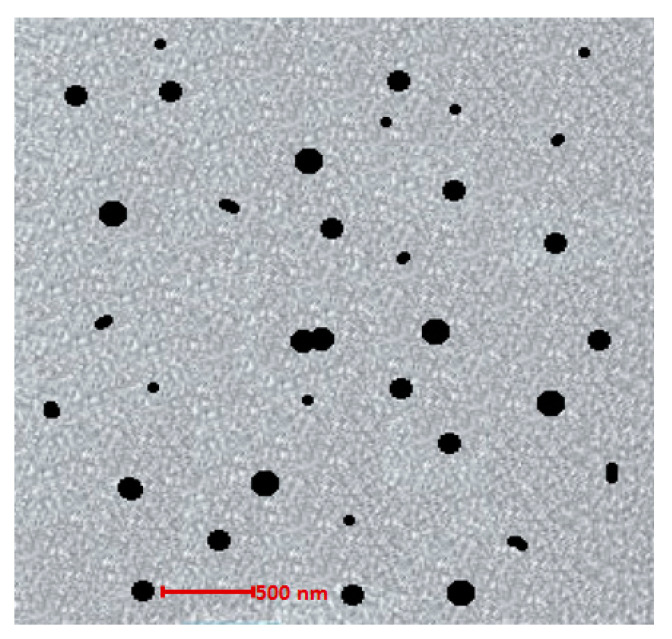
TEM image of optimized nanoemulgel formulation (OMP3NEG).

TEM was utilized to gather crucial morphological data of optimized nanoemulgel formulation (OMP3NEG). The polymeric gel base held nanoemulsion particles that exhibited a spherical shape and homogenous distribution. The gel layer barrier was formed by nanoemulsion particles entrapped in the porous carbopol gel, which allowed for a controlled drug release from the nanoemulgel formulation. Spherical particles of the formulation are advantageous since they readily permeate through tiny skin pores, increasing system permeability [42]. Figure 4 shows the TEM image of the OMP3NEG formulation.

### 3.8. Viscosity Determination

Transdermal or topical drug delivery is largely affected by the rheological behavior of the gel as it affects various parameters such as application convenience, drug release, stability, spreadability, adherence, and retention of formulation at the site of application [33]. A variety of other factors such as the nature of oils, surfactants, co-surfactants, co-solvents, and gelling agents employed in formulation, are also responsible to alter viscosity [43]. Table 3 shows the viscosities of OMP3NE and OMP3NEG formulations, which were measured at various temperatures and time intervals. At a constant shear speed of 40 rpm, the viscosity of OMP3NEG (12,455 ± 14.53 mPa s) was higher as compared to OMP3NE (5889 ± 8.62 mPa s). It could be attributed to the presence of nanoemulsion particles entrapped into the carbopol gel base. In fact, the study by Ullah et al. found that the inculcation of a polymeric gelling agent improves the viscosity of any formulation [44].

### 3.9. Spreadability & Extrudability Estimation

The essential characteristics of topical formulations that determines the therapeutic efficacy and homogenous drug distribution are spreadability and Extrudability [45]. For a formulation to exit a container with the appropriate spreadability, a little shear is needed [46]. The coefficient of spreadability of a topical formulation is impacted by a number of variables, such as higher and lower temperatures. In fact, formulations’ viscosities did indeed rise at low temperatures, decreasing their spreadability. On the other hand, topical formulations’ viscosity is reduced at high temperatures, leading to their considerable spreadability [47]. The values of spreadability and extrudability of OMP3NE and OMP3NEG at various temperatures are shown in Table 4, indicating good spreadability of nano formulations. The spreadability of OMP3NE and OMP3NEG maintained at high temperatures is greater in comparison to those nanoemulsion and nanoemulgel formulations maintained at reduced temperatures. Similarly, both nano formulations possessed excellent extrudability profiles.

### 3.10. In-Vitro Drug Release Evaluation

**Figure 5:**
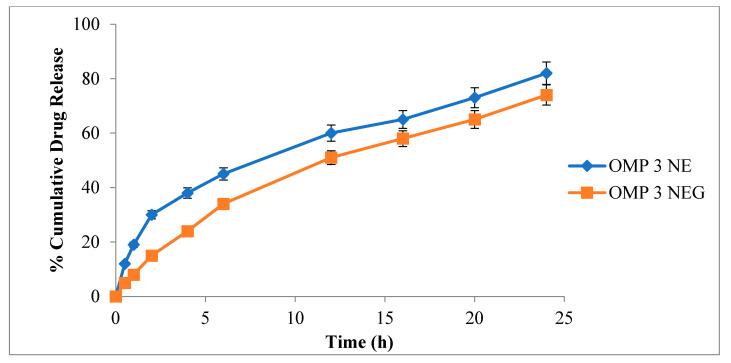
Cumulative drug release (%) from OMP3NE and OMP3NEG at indicated time points (mean ± SD, *n* = 3).

An in-vitro drug release analysis is performed to forecast the reproducibility of drug release duration and rate. For a simulated physiological state of the skin, in-vitro release profiles were determined, as evident in Figure 5. In this process, a receptor medium containing PBS (pH 5.5) kept at 32 ± 0.5 °C was utilized. A comparative analysis between OMP3NE and OMP3NEG was undertaken, and the results are expressed in Figure 3. OMP3NE exhibited fast initial drug release in the first 2 h, followed by continuous release over the next 22 h. The initial burst release of the drug from OMP3NE could be attributed to the superficial trapping of the drug [48]. About 85.28% and 82.16% of the drug are released from OMPNE and OMP3NEG at pH 5.5 after 24 h, respectively. The drug release was observed in the following order with no statistically significant difference (*p* > 0.05) except initial burst release in the case of OMP3NE; OMP3NE > OMP3NEG. The formulation’s nano size improved the rate of drug dissolution into the aqueous phase with a regulated drug release profile [49]. 

### 3.11. Ex-Vivo Permeation

**Figure 6:**
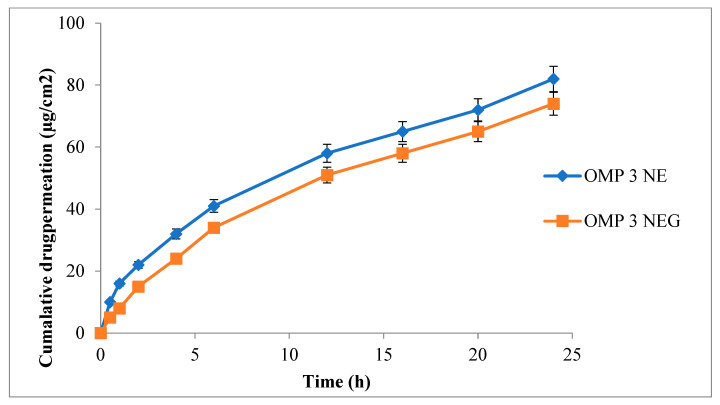
Cumulative drug permeated (μg/cm^2^) from OMP3NE and OMP3NEG at indicated time point (mean ± SD, *n* = 3).

An essential component for effective transdermal drug administration is skin permeation [50]. Both optimized nanoemulsions, as well as nanoemulgel formulations, were evaluated for ex-vivo permeation analysis by employing rabbit skin. Figure 6 demonstrates the percent of cumulative drug permeated from OMP3NE and OMP3NEG. The type of drug, particle size, zeta potential, and surface area are some of the factors that influence transdermal drug permeation [51]. In the current investigation, a Franz diffusion cell was used to assess the drug permeation profile. The permeation of OMP3NE (82.18 ± 1.66 μg/cm^2^) was significantly (*p* < 0.05) higher in comparison to that of OMP3NEG (72.21 ± 1.71 μg/cm^2^). The results of the skin permeation trials show that OMP3NE formulation is crucial for both drug targeting the skin and regulating drug release. Studies have demonstrated that the physicochemical properties of the formulation have an impact on the diffusion rate. These include the capacity for hydrogen bonding, drug loading, surface charge, and drug administration method [52]. Tween 80, employed as a surfactant in the formulation, is responsible for the lipid packing fluidization phenomenon. By using a skin lipid extraction method, it also maximizes the aqueous content of the stratum corneum [53]. Span 80, which is used as a solvent, has an impact on drug permeation from OMP3NE. These cumulative effects cause the epidermis’s barrier functions to be reduced, which enhances transdermal drug delivery. OMP3NE > OMP3NEG was the increased order of skin permeation parameter. Because these formulations may permeate through the skin’s relatively smaller pores, OMP3NE exhibits improved skin penetration.

### 3.12. Microbiological Assay

Once the skin has been injured, external bacteria, as well as the natural flora of the skin, easily get access to the underlying skin tissues and develop colonies in a warm, humid, and enriched environment. Therefore, rapid healing is necessary to prevent the harboring of many bacterial strains [54]. Gram-negative bacteria, such as *Escherichia coli*, *Klebsiella pneumoniae*, and *Pseudomonas aeruginosa* occur in advanced stages of infection that impact deeper skin layers. Gram-positive bacteria, such as *Staphylococcus aureus*, typically appear in the early stages of infection [55]. Therefore, effective control over bacterial growth is needed.

**Figure 7:**
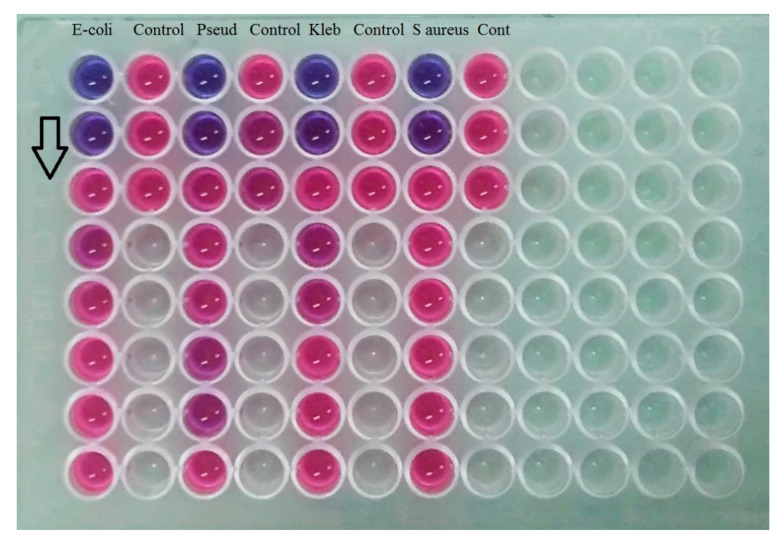
MIC of OMP3NEG against selected bacterial strains using 96 well microplate techniques. The arrow is showing the antimicrobial activity from upward towards downward.

Figure 7 shows the MIC values against the four tested bacterial strains for OMP3NE (control) and the optimized nanoemulgel formulation (OMP3NEG). When compared to OMP3NE (control) with the four bacterial strains, the optimized nanoemulgel had reduced MIC values (*p* < 0.05), as shown in Figure 6. With optimized nanoemulgel formulation, OMP demonstrated a moderate MIC value (1.25 mg/mL) against all tested bacterial strains. The formulation excipients and characteristics of OMP3NEG may be responsible for its improved antibacterial effect. It was previously claimed that the capacity of nanoemulgel formulations to generate tight junctions on the bacterial membrane could increase the antibacterial activity [56]. Additionally, it is widely recognized that unsaturated fatty acids, such as lactic acid, have antibacterial effectiveness because they cause bacterial cell membranes to rupture, causing bacterial lysis [57]. Additionally, the smaller nanoparticles may be the cause of the enhanced antibacterial activity of the optimized nanoemulgel in comparison to the OMP3NE (control) formulation.

### 3.13. Stability Studies

The optimized drug-loaded nanoemulgel formulation (OMP3NEG) had the average particle size evaluated during stability testing in accordance with ICH guidelines. There was no change in the appearance and clarity of the formulation. It was almost identical to how it had been at zero time. During the duration of ambient storage, there was no change in the nanoemulgel’s particle size. However, under accelerated storage circumstances, the nanoemulgel’s particle size rose from 369.7 ± 8.77 nm to 405 ± 9.65, 480 ± 8.87, and 529 ± 9.41 nm at the end of the first, second, and third months, respectively. This demonstrated that as storage temperature was raised, particle size increased. According to stability investigations, the optimized nanoemulgel formulation was stable under typical storage circumstances but clustered when the temperature was raised as shown in Table 5 [58]. 

## 4. Conclusions

The current study demonstrates the fabrication, optimization, and characterization of chitosan-coated omeprazole-based nanoemulgel formulations for topical delivery. the prepared nanoemulgel exhibited a particle size of 369.7 nm and a zeta potential of −15.3 mV. The presence of chitosan in nanoemulgel results in higher entrapment efficiency and controlled release of the drug. Skin drug permeation of gel was slightly lower than nanoemulsion due to the larger size and presence of Carbopol. The prepared nanoemulgel formulation was stable under typical storage circumstances. The efficacy of omeprazole against different bacterial strains was significantly increased when nanoemulgel formulation was used. This was due to formulation ingredients, such as chitosan and the characteristics of nanoemulgel. The most common use of omeprazole is to treat stomach ulcers caused by H pylori, and no studies have been conducted to test its effectiveness as a topical agent for skin infections. The current study depicted that omeprazole showed significant results in treating microbial infections. Further studies should also be recommended to investigate the efficacy of omeprazole using in vivo models.

## Figures and Tables

**Table 1:** Composition of 0.3 % (*w*/*w*) OMP nanoemulsion formulations.

F. Codes	Omeprazole	Olive Oil	Tween 80	Span 80	Distilled Water
Blank NE	-	10	15	3.5	71.5
OMP1NE	0.3	7.5	10	6.5	75.7
OMP2NE	0.3	12.5	13	10	64.2
OMP3NE	0.3	10	15	3.5	71.2

OMP3NE = optimized nanoemulsion formulation.

**Table 2:** Physico-chemical characteristics of blank NE, OMP3NE, and OMP3NEG at 8, 25, and 40 °C.

Formulations	Temperature (°C)	Odor Change	Appearance	Phase Separation	Centrifugation Stability	Thermodynamic Test
Blank NE	8	No	Light brown	No	**	***
	25	No	Light brown	No	**	***
	40	No	Light brown	No	**	***
OMP3NE	8	No	Light brown	No	**	***
	25	No	Light brown	No	**	***
	40	No	Light brown	No	**	***
OMP3NEG	8	No	Light brown	No	**	***
	25	No	Light brown	No	**	***
	40	No	Light brown	No	**	***

** = Good, *** = Passed.

**Table 3:** The viscosity of OMP3NE and OMP3NEG at various temperatures and time intervals.

Day	Viscosity (mPa s)					
	8 °C		25 °C		40 °C	
	OMP3NE	OMP3NEG	OMP3NE	OMP3NEG	OMPNE	OMP3NEG
0	5889 ± 8.62	12,455 ± 14.53	5889 ± 8.62	12,455 ± 14.53	5889 ± 8.62	12,455 ± 14.53
1	5793 ± 7.26	12,299 ± 13.63	5801 ± 8.23	12,366 ± 12.58	5843 ± 7.89	12,378 ± 13.54
2	5710 ± 7.77	12,150 ± 14.01	5745 ± 9.65	12,108 ± 11.47	5796 ± 7.62	12,198 ± 12.49
7	5655 ± 8.11	11,988 ± 13.31	5547 ± 7.62	11,785 ± 11.23	5579 ± 8.41	11,847 ± 12.69
14	5449 ± 8.91	11,929 ± 13.92	5481 ± 8.22	11,547 ± 12.36	5436 ± 9.55	11,785 ± 11.23
28	5385 ± 7.72	11,560 ± 15.21	5300 ± 7.56	11,498 ± 12.84	5317 ± 7.62	11,542 ± 12.82

Note = Data are expressed as mean ± SD (*n* = 3).

**Table 4:** Average spreadability values of OMP3NE and OMP3NEG at various temperatures.

Formulations	Spreadability		
	8 °C	25 °C	40 °C
OMP3NE	18.37 ± 1.09	22.61 ± 1.53	27.33 ± 1.78
OMP3NEG	13.89 ± 1.32	17.31 ± 1.41	21.64 ± 1.59

Note = Data are expressed as mean ± SD (*n* = 3).

**Table 5:** Stability study of OMP3NEG formulation.

Parameters	Temperature	
	4 ± 2 °C	40 ± 2 °C
Particle Size [4]	365.3 ± 6.32	367.1 ± 6.12
PDI	0.313 ± 0.31	0.311 ± 0.13
Zeta Potential (mV)	−15.7 ± 6.1	−15.1 ± 6.5
pH	6.21 ± 0.21	6.12 ± 0.42
Phase Separation	Nil	Nil
Clarity	Transparent and Clear	Transparent and Clear
Drug Content (%) ± SD	90.89 ± 0.12	90.78 ± 0.10
Color Change	No Change	No Change

Data were expressed as mean ± SD; (*n* = 3).

## Data Availability

Not applicable.
